# Circular RNA circAGAP1 promotes sunitinib sensitivity in renal cell carcinoma via sponging multiple PDGFR-targeted miRNAs

**DOI:** 10.32604/or.2024.047698

**Published:** 2025-01-16

**Authors:** QI LV, GANGMIN WANG, YI HONG, TIANYI ZHU, SHUANG QIN, SAIFEI SUN, YUTING WANG, YAOHUA LIU, QING ZHANG, CHUNHUI MA, PEIJUN WANG

**Affiliations:** 1 Imaging Department, Tongji Hospital, Tongji University School of Medicine, Shanghai, 200065, China; 2 Urology Department, Huashan Hospital, Fudan University, Shanghai, 200040, China; 3 Orthopedic Department, Shanghai General Hospital of Shanghai Jiaotong University, Shanghai, 200080, China

**Keywords:** Renal cell carcinoma, Sunitinib resistance, Circular RNA circAGAP1, MiR-149-5p, MiR-455-5p, MiR-15a-5p

## Abstract

**Background:**

Sunitinib resistance is a major challenge in advanced renal cell carcinoma (RCC). Clinically, elucidating the underlying mechanisms and developing practical countermeasures for sunitinib resistance in RCC is desirable. In previous studies, we found that circAGAP1 expression was significantly upregulated in clear cell RCC (ccRCC) and was strongly associated with poor prognosis. However, the role of circAGAP1 in sunitinib resistance in ccRCC remains unclear.

**Methods:**

We used public databases for bioinformatics analysis to identify the binding targets of circAGAP1. Additionally, the effects of circAGAP1 on the proliferation, clonogenesis, apoptosis, and migration of ccRCC cells were analyzed using quantitative real-time PCR, cell counting kit-8 assays, migration and apoptosis assays, and colony formation assays. Furthermore, RNA immunoprecipitation, dual-luciferase reporter, and fluorescence *in situ* hybridization assays were used to explore the molecular mechanism.

**Results:**

In this study, circAGAP1 exhibited higher expression in sunitinib-sensitive ccRCC cells and inhibited the clonogenesis, proliferation, and migration of ccRCC cells after sunitinib treatment. Mechanical studies revealed that circAGAP1 regulated the expression of sunitinib target platelet-derived growth factor receptor by acting as a microRNA sponge that suppresses miR-149-5p, miR-455-5p, and miR-15a-5p simultaneously. Overexpression of these three miRNAs reversed circAGAP1-mediated sunitinib sensitivity in ccRCC.

**Conclusions:**

In summary, our findings indicate that circAGAP1 may serve as a promising biomarker to predict sunitinib sensibility and a therapeutic target in ccRCC.

## Introduction

Renal cell carcinoma (RCC), commonly referred to as kidney cancer [1], is marked by a distressing escalation in its incidence rate, a trend that persists every year [2,3]. Clear cell RCC (ccRCC) is the most prevalent subtype of RCC, accounting for 75–80% of cases [1–4]. Notably, a substantial 20–30% of patients are diagnosed with advanced-stage RCC, often precluding the opportunity for timely surgical intervention or appropriate treatment initiation [5]. Sunitinib, among the primary therapeutic agents employed in the management of advanced RCC, has demonstrated commendable efficacy [6–8]. It is a receptor tyrosine kinase inhibitor that inhibits angiogenesis and RCC progression mainly by inhibiting the activities of platelet-derived growth factor receptor (PDGFR), vascular endothelial growth factor receptor (VEGFR) and receptor tyrosine kinase [9].

However, as the duration of sunitinib treatment increases, a significant proportion of patients inevitably develop resistance to the drug, thereby limiting its clinical utility [10]. Recent studies on sunitinib resistance have aimed to uncover genetic mutations and signaling pathway alterations that contribute to drug resistance [11–13]. Concurrently, we also paid attention to the role of circular RNAs (circRNA) in tumor resistance. Studies have focused on the role of drug efflux pumps and the effect of the tumor microenvironment in promoting resistance. Advances include exploring combination therapies, next-generation drugs, and nanotechnology for better drug delivery, with the aim of improving treatment outcomes and tackling resistance in cancer therapy.

**Figure A1:**
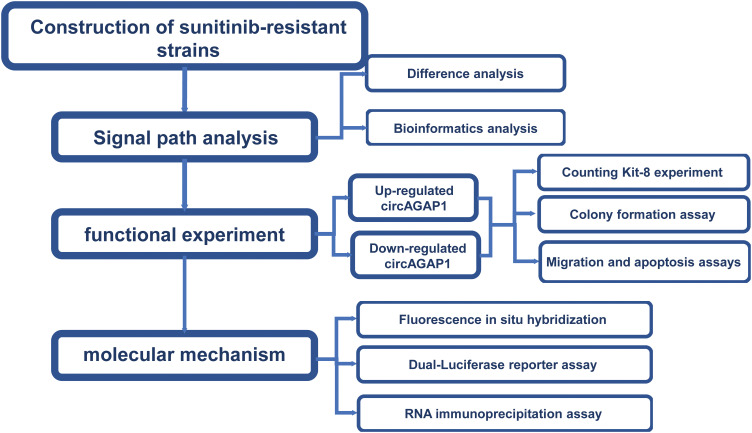
A flowchart.

Notably, circRNAs are widely dispersed throughout the body and have vital biological activities [14–17], and are involved in tumor chemoresistance through various mechanisms of action [16–20], including in kidney cancer. For instance, CircME1 promotes sunitinib resistance in ccRCC through the cis-regulation of ME1 [21]. In addition, circRNAs can act as “sponges” for microRNAs (miRNAs), reducing their ability to target mRNAs [22–24] and driving anti-PD-1 resistance [25]. Based on our previous studies, several novel circRNAs specific to RCC have been successfully identified and two studies have been published [26,27]. Therefore, circAGAP1 may serve as a potential target for ccRCC treatment and predictor of sunitinib sensitivity. We discovered that circAGAP1 expression was considerably upregulated in ccRCC and was closely associated with poor prognosis. Furthermore, circAGAP1 was highly expressed in sunitinib-sensitive ccRCC cells and influenced their proliferation and clonogenic abilities. Further research indicates that circAGAP1 regulates the expression of the sunitinib target PDGFR by acting as an miRNAs sponge. A flowchart (Fig. A1) has been added to illustrate the research methodology. Based on our results, circAGAP1 may serve as a promising biomarker for predicting sunitinib sensitivity and therapeutic target for ccRCC.

## Materials and Methods

### Cell lines and cell culture

We sourced human ccRCC cell lines (786-O, ACHN, A498, OS-RC-2, and caki-2) and 293T cells from the Shanghai Academy of Sciences, China. To develop ccRCC cell lines with resistance (notably 786-O-ST and ACHN-ST), we gradually increased the concentrations of the drug used. These cells were cultured in a modified version of Dulbecco’s Modified Eagle Medium (catalog number: KGM12800N-500, keygen biotech, Jiangsu, China), provided by KeyGEN BioTECH, based in Jiangsu, China, supplemented with serum (catalog number: B7447-1000, Sigma-Aldrich, USA) and antibiotics (catalog number: KGY0023, Keygen Biotech, Jiangsu, China). Conditions within the incubator were carefully controlled, ensuring a constant temperature of 37°C and an atmosphere containing 5% CO_2_.

### Transfection

Human circAGAP1 (hsa_circ_0058792) plasmid was synthesized by Ruibo (Guangzhou, China). GenePharma (Shanghai, China) supplied miR-149-5p, miR-455-5p, and miR-15a-5p mimic and matched controls (NC). Lipofectamine 2000 (catalog number: lipo2000:11668019, Invitrogen, USA) was used to transfect plasmids, small interfering RNAs (siRNAs), mimics, or inhibitors of miR-149-5p (mimic sense: uctggcuccgugucuucacuccc; antisense: gggagugaagacacggagccaga), miR-455-5p (mimic sense: UAUGUGCCUUUGGACUACAUCG; antisense: CGAUGUAGUCCAAAGGCACAUA), miR-15a-5p (mimic sense: UAGCAGCACAUAAUGGUUUGUG; antisense: CACAAACCAUUAUGUGCUGCUA). The following siRNA sequences were used: circAGAP1 siRNA (5′GACGATGCCTTCGTGAACA-3′) and control siRNA (5′-TTCTCCGAACGTGTCACGT-3′).

### Sunitinib resistance induction

Inducing resistance to sunitinib involved continuously exposing cells to gradually increased doses of sunitinib (free base, supplied by LC Laboratories in Woburn, MA, USA), continuing until they could tolerate the maximum dose achievable. Specifically, 786-O cells underwent treatment with sunitinib doses that varied from 3 to 7 µM, whereas doses for ACHN cells ranged from 0.5 to 4 µM. As benchmarks, we utilized therapy-responsive (original) cell lines of both 786-O and ACHN. The determination of resistance in both 786-O and ACHN cell varieties was made when their half-maximal inhibitory concentrations, after receiving sunitinib for 72 h, increased by approximately 4 times.

### Quantitative real-time PCR (qRT-PCR)

Using the TRIzol reagent (catalog number: 15596026CN, Thermo Fisher, USA) by Invitrogen in Carlsbad, CA, USA, we performed total RNA extraction from a variety of kidney carcinoma cells, including 786-O, ACHN, A498, and ACHN-ST, 786-O-ST cells. Following the guidelines provided by the manufacturer, this RNA was then converted back into complementary DNA (cDNA) with the assistance of the PrimeScript RT Reagent Kit (catalog number: RR036B, TaKaRa, Dalian, China) Reagent Kit, which includes a gDNA Eraser, sourced from TaKaRa in Dalian, China. A primer’s specificity was verified through the generation of a melting curve. DNA amplification was performed for 40 cycles with 15 s of denaturation at 95°C, followed by 60 seconds of annealing at 60°C. The data generated from each PCR reaction were analyzed using the *t*-test, based on the 2^−▵▵Ct^ calculation method. For normalization purposes, beta-actin gene expression served as the internal standard, facilitating the comparison with the average expression levels (mean ± SD) of circRNA and miRNA.

The following primers were used: circAGAP1-F, 5′-GTCTTCCAGGACGATGCCTT-3′; circAGAP1-R, 5′-GCTGGCCAAGTTACCCACAA-3′; hsa-miR-15a-5p-F, 5′-ACTCCAGGGCTACAACTGGTCGTGGAGTCGGCAATTCAGTTGAGCACAAACC-3′; hsa-miR-15a-5p-R, 5′-ACACTCCAGGGCGGCAACTGGTGTCGCAAT-3′; has-miR-149-5p-F, 5′-CCTCTGGCTCCGTGTCTTC-3′; has-miR-149-5p-R, 5′-CAGTGCAGGGTCCGAGGT-3′; has-miR-455-5p-F, 5′-GGGCTATGTGCCTTTGGAC-3′; has-miR-455-5p-R, 5′- CAGTGCAGGGTCCGAGGT-3′; PDGFR-F, 5′-GCAGGAGTTTGAGGTGGTGAGC-3′; and PDGFR-R, 5′-AAAGGGCAAGGAGTGTGGCAC-3′.

### Cell viability assessment utilizing the cell counting kit-8 (CCK-8) method

Cells were allocated into 96-well microplates (manufactured by NEST, Jiangsu, China) at a uniform distribution of 1000 cells per well. Following initial seeding, cells subjected to CircAGAP1 knockdown underwent transfection with either miRNA inhibitors or a non-coding miRNA control (miR-NC). In parallel, cells exhibiting circAGAP1 overexpression received transfection treatments with miRNA mimics or miR-NC 12 h subsequent to seeding. Thereafter, a volume of 10 µL of CCK-8 solution (catalog number: CK04, provided by Dojindo, Japan) was dispensed into each well, subsequent to which the cells were incubated under dark conditions for a period of 2 h. Optical density (OD) measurements at a wavelength of 450 nm were systematically acquired using a Bio-Tek Flx800T Fluorescence reader (FLX800T, Biotech, USA) at daily intervals from day 1 through day 5 post-seeding.

### Migration and apoptosis assays

In the current study, we performed migration and apoptosis assays as previously described [27]. Briefly, for the migration assay, the cells were seeded in 24-well plates and allowed to reach 80% confluence before a scratch was formed. Cell migration to the scratched area was monitored and quantified after 24 h. Apoptosis was assessed using Annexin V/PI staining (catalog number: 556547, BD, USA), followed by flow cytometry. For detailed methods, please refer to our previous study [27].

### Assay for colony formation

Subsequent to transfection procedures, cells underwent digestion with 0.25% trypsin (sourced from Solarbio, Beijing, China) and were subsequently re-plated in six-well plates at a seeding density of 300 cells per well. Following a 14-day incubation period, cells were fixed using methanol and stained with both crystal violet and paraformaldehyde for a duration of 30 min. Finally, the cells were observed under a microscope (IX73, Olympus, Japan) and the number of clones formed was counted. A total of three independent experiments were performed.

### RNA immunoprecipitation (RIP) assay

The RIP assay was executed employing the EZ-Magna RIP RNA Binding Protein Immunoprecipitation Kit (catalog number: 17-701, Milipore, USA) to ascertain the association of miR-149-5p, miR-455-5p, and miR-15a-5p with has_circ_AGAP1. In brief, ACHN cells were lysed using a specialized RIP buffer (catalog number: SCR-2, gzscbio.com, Guangzhou, China) immediately after being collected. The lysates were then incubated overnight at 4°C with magnetic beads bound to either anti-Ago2 antibodies or IgG. Following this, RNA that had been immunoprecipitated was extracted and subjected to purification, in preparation for analysis via qRT-PCR.

### Dual-luciferase reporter assay

The assessment of luciferase reporter activity was conducted using dual-luciferase reporter vectors acquired from Ruibo (Guangzhou, China). Preliminary to transfection, 293T cells were plated in 24-well plates (2.5 × 10^4^) for 12 h. Constructs of pmirGLO-circAGAP1-wild type (WT) and pmirGLO-circAGAP1-mutant (Mut), alongside the pRL-TK vector (Promega, Madison, WI, USA), were co-transfected with mimics of miR-149-5p, miR-15-5p, and miR-455-5p or a control mimic (mimic NC) using Lipofectamine 2000 (Invitrogen). After a 48-h transfection period, the culture medium was removed, and cells were cleansed with PBS. The dual-luciferase assay kit (Promega) (catalog number: E1910, USA) was employed to measure luciferase activity in accordance with the manufacturer’s protocol. All experimental iterations were conducted in triplicate to ensure data validity.

### Fluorescence in situ hybridization (FISH)

Notably, FITC-labeled hsa-circAGAP1 and cy3-labelled probes targeting miR-149-5p, miR-455-5p, and miR-15a-5p were designed. A Fluorescent *In Situ* Hybridization Kit (catalog number: C10910, RIBBIO, Guangzhou, China) was used for this experiment. Briefly, ACHN cells were prepared on slides and fixed. Hybridization was performed according to the manufacturer’s instructions. DAPI-labelled 18S RNA probes (catalog number: 18S rRNA Cy3 FISH Probe, R0312, Beyotime, Jiangsu, China) were used to label the nuclei. Finally, images were acquired using a confocal fluorescence microscope (LSM 710, ZEISS, Germany).

### Bioinformatics analysis

Public databases, such as the circinteractome and cancer-specific circRNA, were used to predict the miRNA targets of circAGAP1. Using TargetScan (http://www.targetscan.org/) and miRDB (http://mirdb.org/), 13 PDGFR-targeted miRNAs and the binding sites of these miRNAs and circAGAP1 were predicted.

### Statistical analysis

Statistical graphs were generated using GraphPad Prism 10 software (GraphPad Software Inc., Boston, MA, USA). A two-tailed Student's *t*-test and one-way ANOVA were used to analyze the corresponding data with each assay performed in triplicate (n = 3). A two-tailed *p*-value of less than 0.05 was considered to indicate a statistically significant difference.

## Results

### CircAGAP1 functions as a super circRNA that regulates the sensitivity of sunitinib therapy in ccRCC

**Figure 1:**
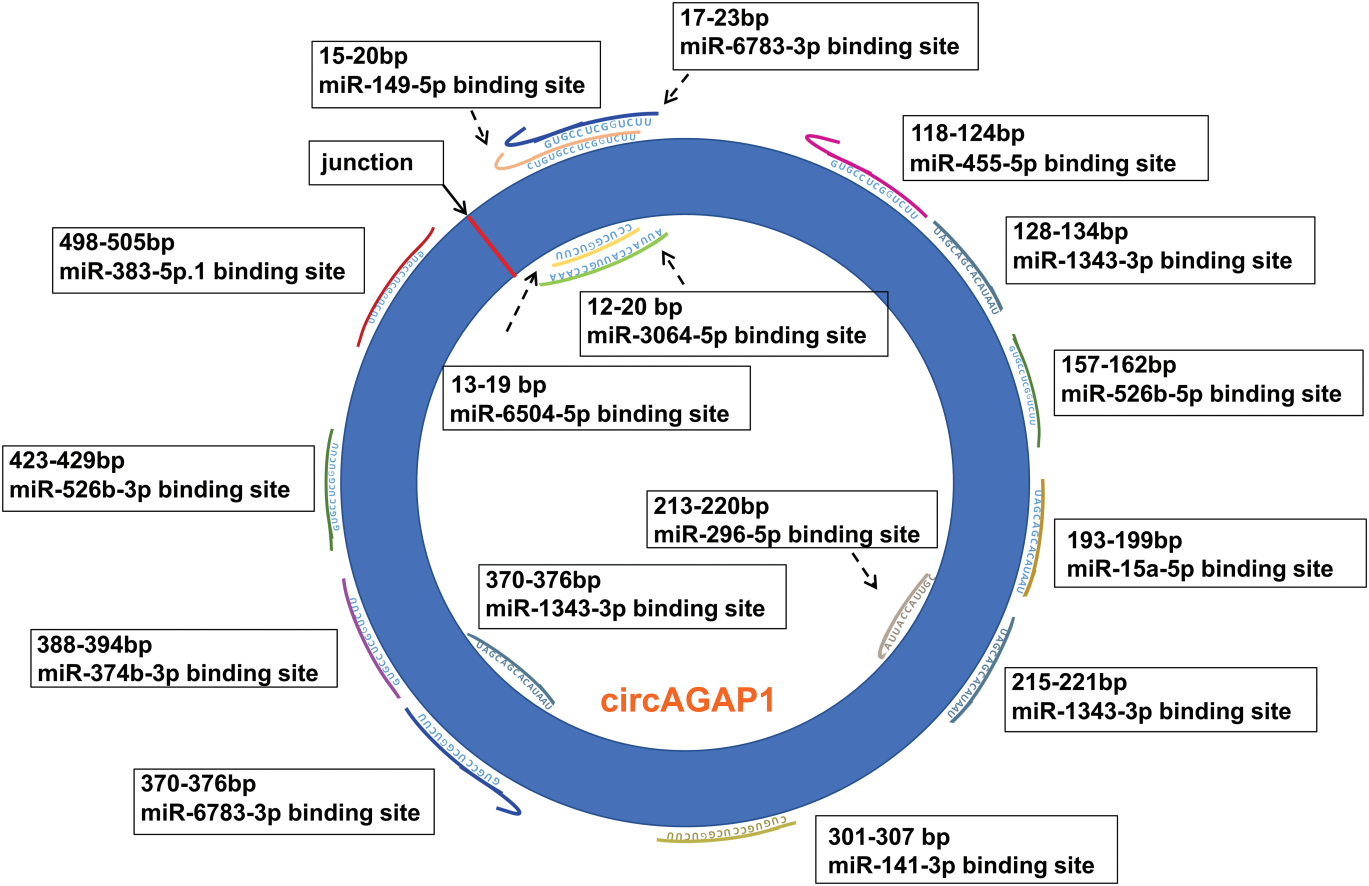
The binding sites of circAGAP1 that adsorb sunitinib target-associated miRNAs.

By combining the properties of circAGAP1, we first used databases to predict multiple miRNA targets of circAGAP1 associated with the targets of sunitinib. According to the TargetScan database, up to 13 miRNAs were found to have mRNA binding sites of PDGFRA (encoding PDGFRα), PDGFRB (encoding PDGFRβ), FLT1 (encoding VEGFR-1), and KdR (encoding VEGFR2), with most having more than one binding sites. Therefore, these 13 miRNAs were collectively referred to as miR-X, and their site information is shown in Fig. 1. This suggests that circAGAP1 may be a super circRNA that regulates sensitivity to sunitinib therapy.

### Effects of circAGAP1 knockdown on the proliferation and clone formation ability of sunitinib-treated ccRCC

**Figure 2:**
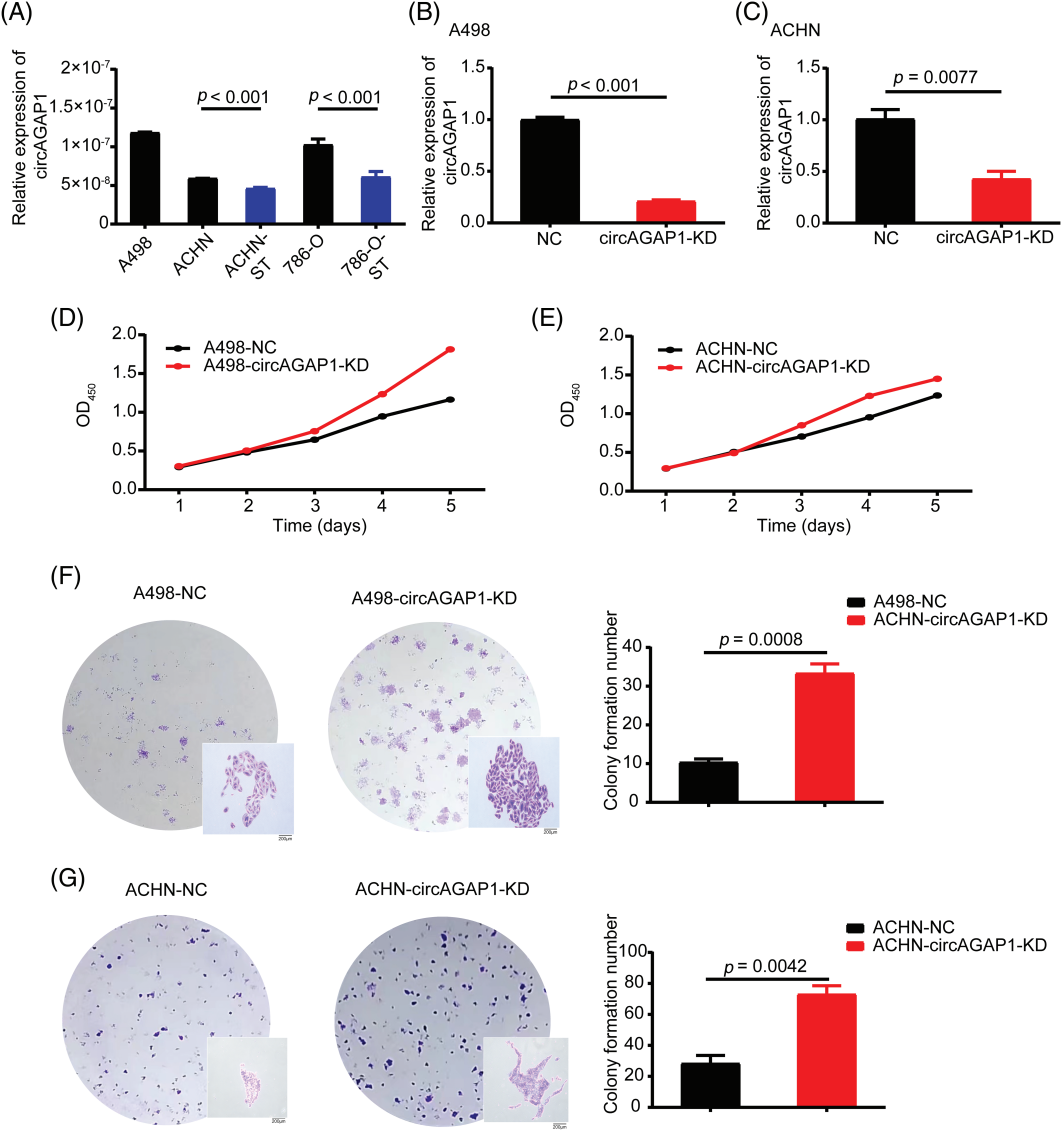
The expression of circAGAP1 and its effect on sunitinib sensibility. (A) Relative expression of circAGAP1 in sunitinib-sensitive (A498, ACHN, 786-O) and drug-resistant cell lines (ACHN-ST, 786-O-ST). (B, C) Detection of the circAGAP1 expression levels in circAGAP1 knockdown cell lines. (D, E) CCK-8 assay of circAGAP1-knockdown and control A498 and ACHN cells with sunitinib treatment at indicated concentrations of 1.5 μM. (F, G) Colony formation assay of circAGAP1-knockdown and control A498 and ACHN cells with sunitinib treatment.

**Figure A2:**
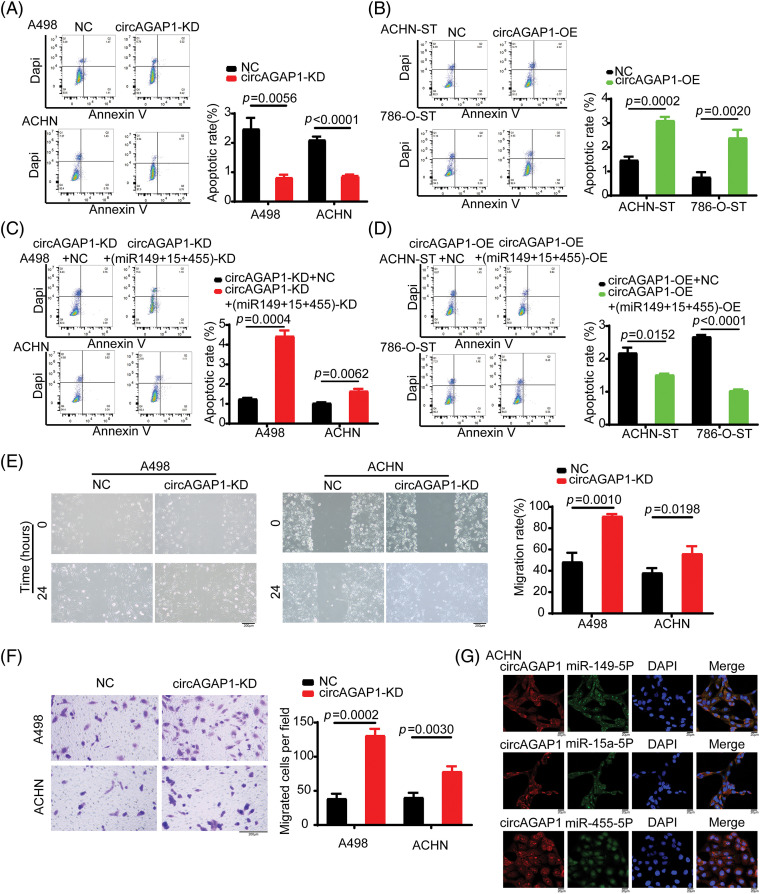
Additional experiments. (A) Flow cytometric analysis detects apoptosis cell proportion with/without circAGAP1 knockdown in A498 and ACHN cells with sunitinib treatment. (B) Flow cytometric analysis detects apoptosis cell proportion with/without overexpression of circAGAP1 in ACHN-ST and 786-O-ST cells with sunitinib treatment. (C, D) Flow cytometric analysis detects apoptosis cell proportion in miRNAs and circAGAP1 knockdown or overexpression cells (A498, ACHN, ACHN-ST and 786-O-ST) and control group cells with sunitinib treatment. (E, F) Cell migration and transwell assays evaluate the migration ability of A498 and ACHN cells treated with sunitinib. (G) The subcellular location of miR-149-5p, miR-455-5p, miR-15-5p and circAGAP1 were demonstrated by FISH assay. Scale bars, 20 μM.

We investigated the role of circAGAP1 in predicting sunitinib sensitivity in renal carcinoma. First, we successfully induced drug-resistant renal cancer cells (786-O-ST and ACHN-ST) by increasing the drug concentration. Compared to the parental cell lines, 786-O-ST and ACHN-ST cells exhibited a poor response to sunitinib. Furthermore, 786-O-ST and ACHN-ST cells could divide freely in media containing 7 or 4 μM sunitinib, respectively. In addition, 498 and ACHN cells are sunitinib-sensitive cell lines, and these two sensitive cell lines were selected during the functional experiment. Subsequently, we detected circAGAP1 expression in parental renal cancer and drug-resistant RCC. Among the tested cell lines, sunitinib-sensitive cells (ACHN and 786-O cells) showed high circAGAP1 expression (Fig. 2A). To investigate the functional relevance of circAGAP1 in sunitinib sensitivity, we stably knocked down circAGAP1 in A498 and ACHIN cells (Figs. 2B and 2C) by constructing shRNAs against circAGAP1 and observed the proliferation and clone formation ability of the cells after sunitinib treatment. The CCK-8 experiment demonstrated that the proliferation of A498 and ACHN cells increased following circAGAP1 knockdown compared to that in control cells (Figs. 2D and 2E). After the suppression of circAGAP1, the clonogenic capacity of A498 and ACHN cells was enhanced upon sunitinib treatment (Figs. 2F and 2G), suggesting that circAGAP1 knockdown weakened the sunitinib response in RCC. Moreover, flow cytometric analyses showed that the proportion of apoptotic cells significantly decreased when circAGAP1 was knocked down in both cell lines (Fig. A2(A)). The knockdown of circAGAP1 partially abrogated the activation of apoptosis in A498 and ACHN cells. A noticeable increase in the migratory abilities of A498 and ACHN cells was observed when circAGAP1 was silenced (Figs. A2(E) and A2(F)). These findings suggest that circAGAP1 is indispensable for sunitinib sensitivity in ccRCC.

### Effects circAGAP1 overexpression on the proliferation and clone formation ability of sunitinib-resistant ccRCC

**Figure 3:**
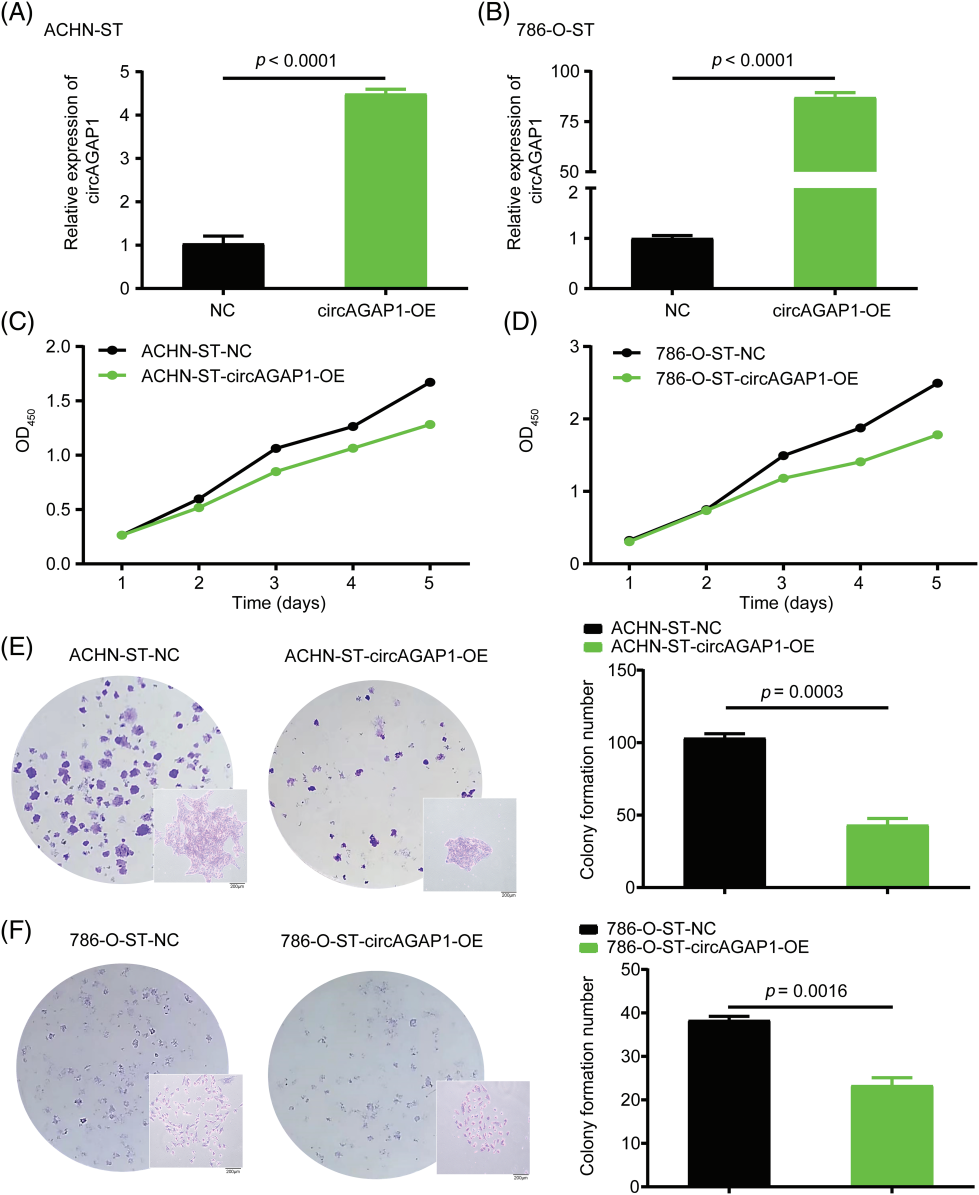
Overexpression of circAGAP1 weakens the proliferation and clone formation ability of sunitinib-resistant ccRCC. (A, B) Detection of the circAGAP1 expression levels in circAGAP1 overexpression cell lines. (C, D) CCK-8 assay of circAGAP1-overexpression and control ACHN-ST and 786-O-ST cells with sunitinib treatment at indicated concentrations of 1.5 μM and 4 μM, respectively. (E, F) Colony formation assay of circAGAP1-overexpression and control ACHN-ST and 786-O-ST cells with sunitinib treatment.

To confirm the role of circAGAP1 in sunitinib efficacy, we generated ACHN-ST and 786-O-ST cells overexpressing circAGAP1 (Figs. 3A and 3B). The results of the CCK-8 and colony formation assays showed that the proliferation and clone formation abilities of ACHN-ST and 786-O-ST cells were decreased in the circAGAP1-overexpressing group compared to the control group (Figs. 3C–3F). Moreover, the rate of apoptosis detected by flow cytometry demonstrated that the percentage of apoptotic events significantly increased when circAGAP1 was overexpressed in sunitinib-resistant cells with sunitinib treatment (Fig. A2(B)). Overall, these findings demonstrate that sunitinib sensitivity increases dramatically following circAGAP1 overexpression.

### CircAGAP1 acts as a molecular sponge for multiple miRNAs

**Figure 4:**
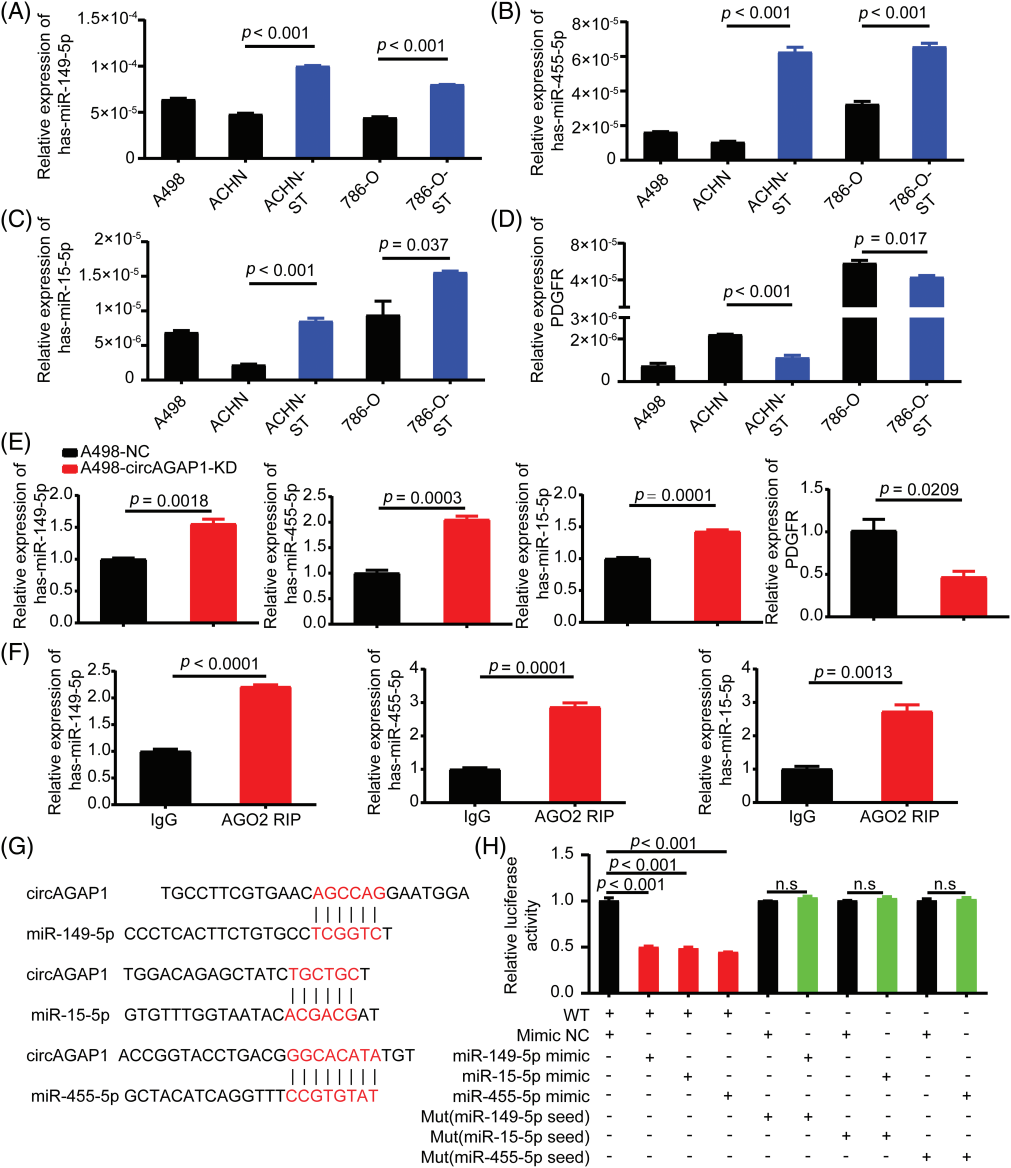
CircAGAP1 targets miR-149-5p, miR-455-5p, and miR-15-5p, and regulates the expression of sunitinib target PDGFR. (A–D) Expression of miR-149-5p, miR-455-5p, miR-15a-5p and target PDGFR in sunitinib-sensitive (A498, ACHN, and 786-O) and drug-resistant cell lines (ACHN-ST and 786-O-ST). (E) Expression of miR-149-5p, miR-455-5p, miR-15a-5p and target PDGFR in A498 and ACHN cells after circAGAP1 knockdown. (F) RIP experiments verifying the binding between miR-149-5p, miR-455-5p, miR-15a-5p, and circAGAP1. (G) Sequence alignment of wild-type (WT) circAGAP1 with miR-149-5p, miR-15a-5p, and miR-455-5p, with mutant (MUT) seed regions highlighted in red. (H) Luciferase activity of WT and MUT circAGAP1 after co-transfection with miRNA mimics (NC, miR-149-5p, miR-15a-5p, miR-455-5p). WT constructs showed significantly reduced activity (*p* < 0.001), while MUT constructs showed no significant changes (n.s.). Data are presented as mean ± SD (n = 3).

We previously discovered that circAGAP1 functions as an miRNA sponge and influences gene expression [27]. Therefore, we predicted the potential target miRNAs through bioinformatics analysis and identified 13 candidate miRNAs that could bind to circAGAP1 (Fig. 1). We then measured miRNA expression in sunitinib-sensitive (A498, ACHN, and 786-O) and drug-resistant cell lines (ACHN-ST and 786-O-ST) and discovered that miR-149-5p, miR-455-5p, and miR-15-5p were significantly downregulated in sunitinib-sensitive cell lines compared with drug-resistant cell lines, with the results being statistically significant (Figs. 4A–4C). In contrast, PDGFR expression was significantly upregulated in the sunitinib-sensitive cell lines (Fig. 4D). In addition, the FISH assay indicated that miR-149-5p, miR-455-5p, miR-15-5p and circAGAP1 were located mainly in the cytoplasm (Fig. A2G). After circAGAP1 knockdown in A498 cells, the expression of miR-149-5p, miR-455-5p, and miR-15a-5p markedly increased, whereas that of the target gene PDGFR significantly decreased (Fig. 4E). The binding between miR-149-5p, miR-455-5p, miR-15a-5p, and circAGAP1 was verified using RIP experiments in ACHN cells (Fig. 4F).

To verify the relationship between miR-149-5p, miR-455-5p, miR-15a-5p and circAGAP1, we constructed a dual-luciferase reporter gene detection system. We constructed circAGAP1 fragments with wild-type (wt) or mutant (mut) complementary binding sites. We co-transfected luc-circAGAP1-wt or luc-circAGAP1-mut plasmid with miR-149-5p mimics, miR-455-5p mimics, miR-15a-5p mimics, or a negative control (NC) into 293T cells. Dual-luciferase reporter gene experiments confirmed that the luciferase activity of circAGAP1-wt was significantly reduced when co-transfected with miR-149-5p, miR-455-5p, or miR-15a-5p mimics than that in the NC group. However, in the circAGAP1 mutant group, luciferase activity in the miR-149-5p, miR-455-5p, and miR-15a-5p mimic transfer groups did not significantly change compared to that in the NC group (Figs. 4G and 4H). Taken together, these results show that circAGAP1 can bind to multiple miRNAs, including miR-149-5p, miR-455-5p, and miR-15a-5p, and regulate the expression of the sunitinib target PDGFR.

### miRNAs (miR-149-5p, miR-455-5p, miR-15a-5p) were replenished or inhibited in circAGAP1 knockdown or overexpressed RCC cells

**Figure 5:**
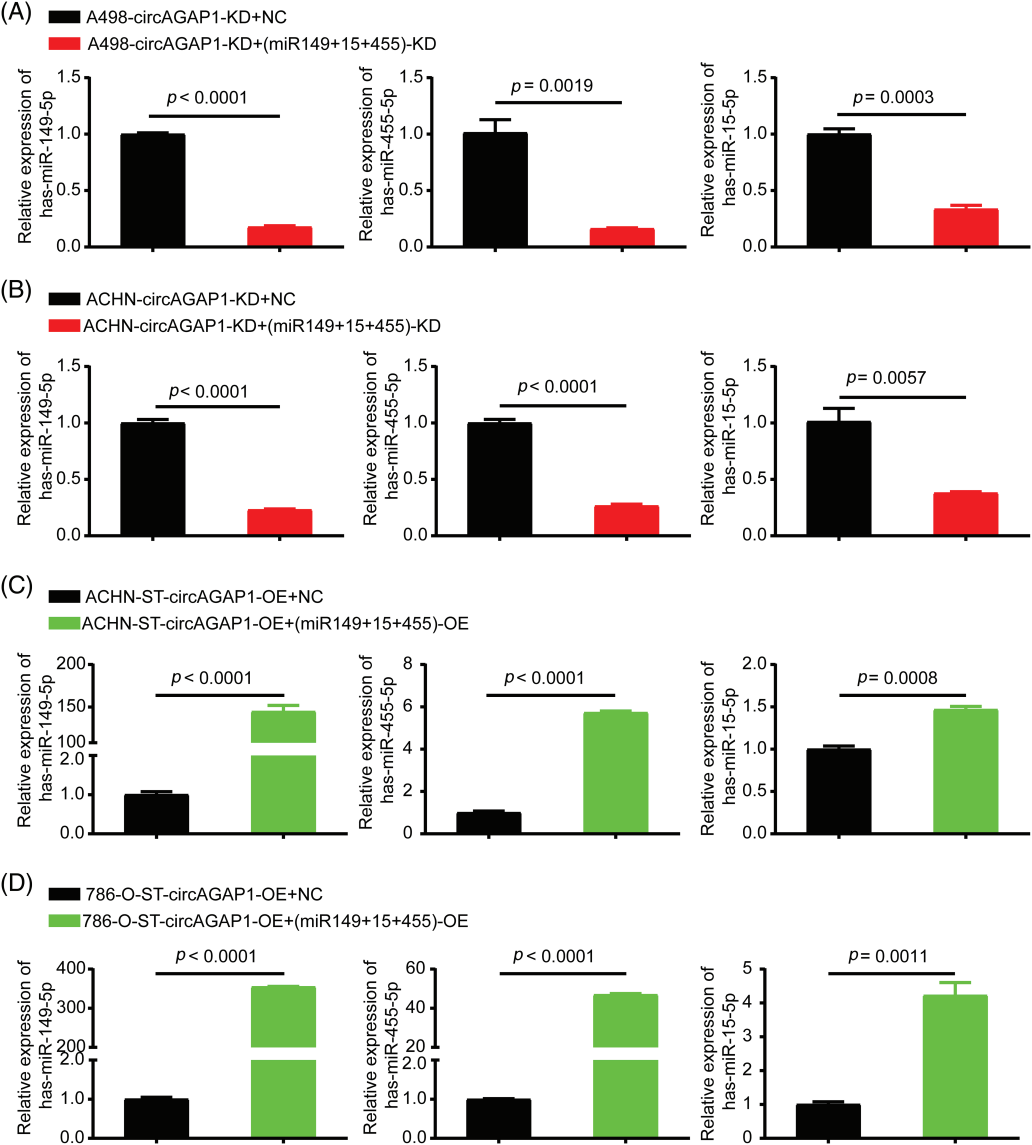
MiR149-5p, miR455-5p, and miR15-5p interference or remediation lines were identified in circAGAP1 knockdown or overexpressed cells. (A, B) Cell lines having miRNA (miR-149-5p, miR-455-5p, and miR-15a-5p) interference with circAGAP1 knockdown in sunitinib-sensitive cell lines (A498 and ACHN). (C, D) Overexpression of circAGAP1 and miRNAs (miR-149-5p, miR-455-5p, and miR-15a-5p) in sunitinib-resistant cell lines (ACHN-ST and 786-O).

The ability of miRNAs (miR-149-5p, miR-455-5p, and miR-15-5p) to bind to circAGAP1 was confirmed. Further functional experiments are required to confirm whether these miRNAs are involved in the mechanism by which circAGAP1 affects ccRCC susceptibility to sunitinib. Therefore, we constructed miRNA (miR-149-5p, miR-455-5p, and miR-15-5p) interference and remediation cell lines in circAGAP1 knockdown and overexpressing cells, respectively. Cells from the miRNA knockdown group expressed lower levels of miR149-5p, miR455-5p, and miR15-5p than those from the control group (Figs. 5A and 5B). In addition, cells from the miRNA overexpression group expressed higher levels of miR-149-5p, miR-455-5p, and miR-15-5p than those in the control group (Figs. 5C and 5D).

### Knockdown or overexpression of miRNAs affected the proliferation and clone formation ability of RCC after sunitinib treatment

**Figure 6:**
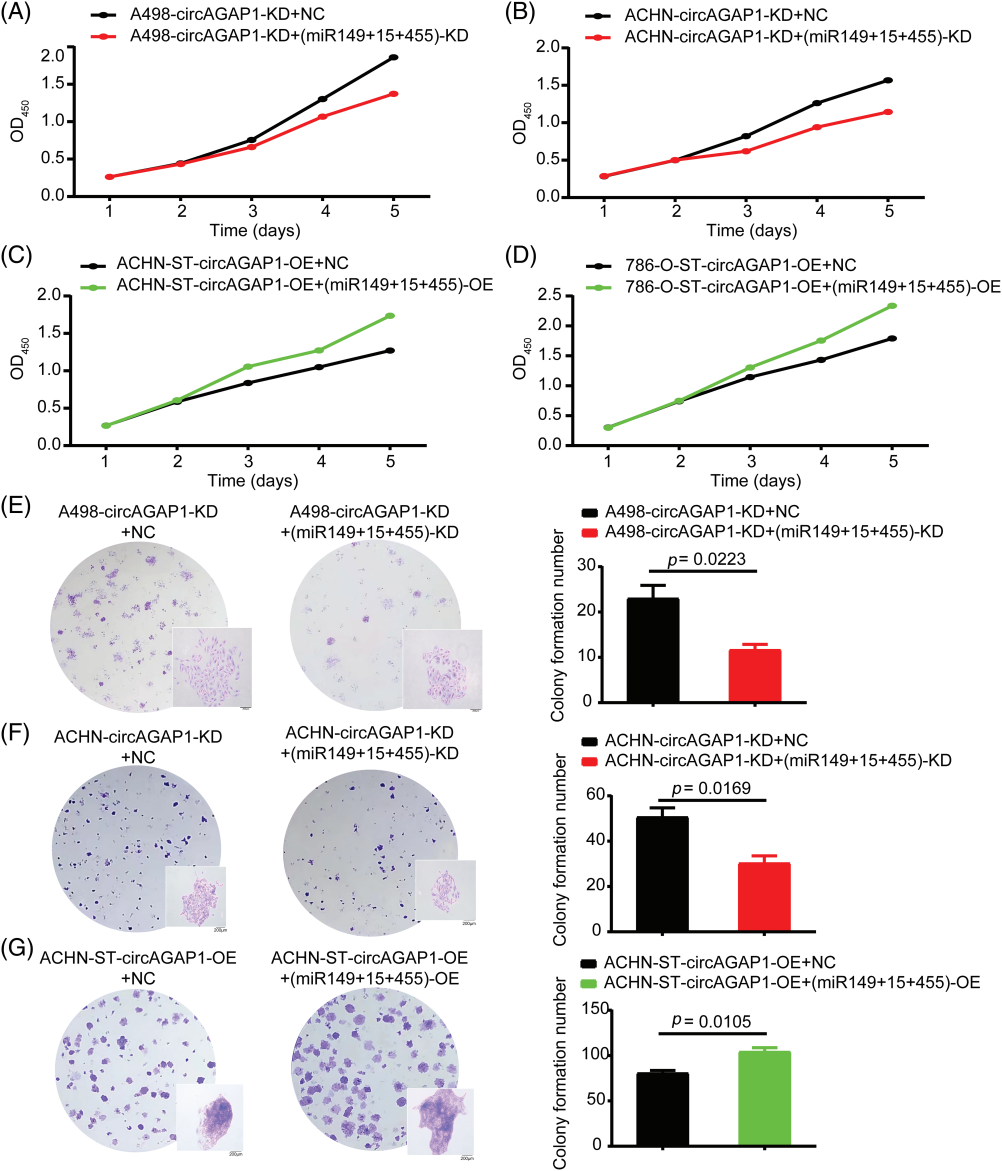
After interference or overexpression of miR-149-5p, miR-455-5p, and miR15-5p in circAGAP1 knockdown or overexpression cells, the proliferation and clone formation abilities of ccRCC cells were reversed. (A–D) CCK-8 assay of interference or overexpression of miRNAs (miR-149-5p, miR-455-5p, and miR-15a-5p) in circAGAP1 knockdown or overexpression (A498, ACHN, and 786-O-ST) and control group cells with sunitinib treatment. (E–G) Colony formation assay of interference or overexpression of miRNAs (miR-149-5p, miR-455-5p, and miR-15a-5p) in circAGAP1 knockdown or overexpression (A498 and ACHN) and control group cells with sunitinib treatment at indicated concentrations of 1.5 μM.

**Figure 7:**
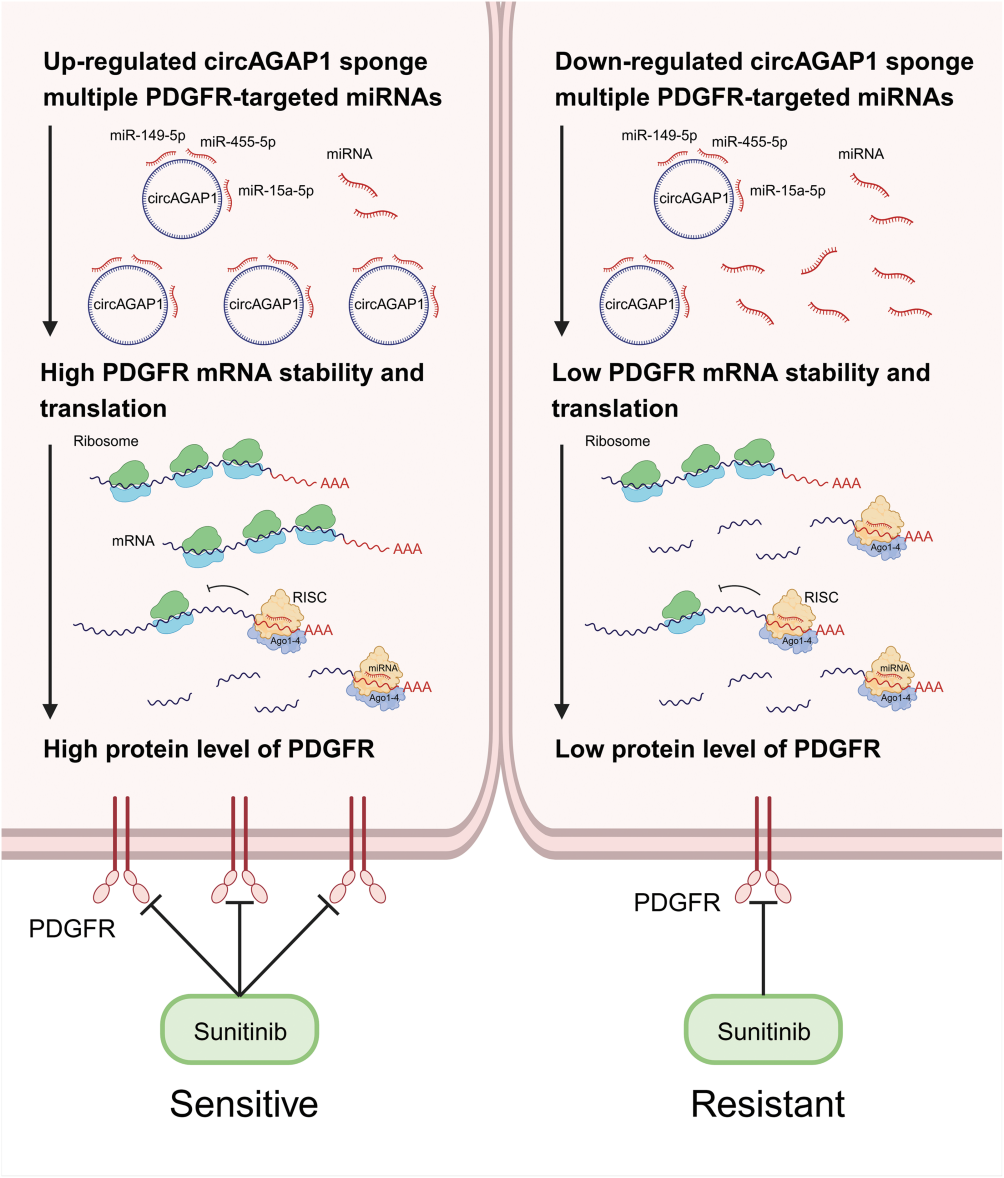
CircAGAP1 promotes sunitinib sensitivity in ccRCC via sponging multiple PDGFR-targeting miRNAs.

After successfully constructing miRNA (miR-149-5p, miR-455-5p, and miR-15-5p) interference and remediation lines, we investigated whether circAGAP1 plays a role in sunitinib efficacy through these miRNAs. The CCK-8 and colony formation assays demonstrated that the proliferation and clone formation of the miRNA-knockdown group were weakened than those of the NC group (Figs. 6A, 6B, 6E and 6F). Furthermore, the apoptosis rate of circAGAP1 knockdown cells was significantly increased when miRNAs were interfered with in flow cytometry analyses (Fig. A2(C) and A2(D)). Overexpression of circAGAP1 weakened the proliferation and clone formation abilities of ACHN-ST cells after sunitinib treatment; however, the proliferation and clone formation of RCC cells increased after the addition of miR-149-5p, miR-455-5p, and miR-15-5p mimics (Figs. 6C, 6D, and 6G). Moreover, overexpression of miRNAs reversed the apoptosis-promoting function of circAGAP1 following sunitinib treatment. These results showed that knockdown or overexpression of miRNAs can affect the proliferation and clone formation abilities of circAGAP1 knockdown or overexpression cells after sunitinib treatment (Fig. 7).

## Discussion

Sunitinib has emerged as one of the primary first-line drugs for RCC treatment, demonstrating significant efficacy in the management of this aggressive cancer [28]. However, a recurring concern in the clinical setting is the development of varying degrees of resistance to sunitinib with prolonged use. This phenomenon underscores the urgent need to elucidate the mechanisms underlying sunitinib resistance. We aimed to elucidate the molecular and cellular processes responsible for this resistance. By gaining a comprehensive understanding of these mechanisms, we hope to identify novel therapeutic targets and strategies that can either prevent or delay the onset of sunitinib resistance, and ultimately improve the prognosis and quality of life of patients with RCC. Our study underscores the importance of continuous research efforts in oncology to advance the frontiers of cancer treatment.

Notably, circRNAs are a class of non-coding RNAs that have gained significant attention owing to their roles in various cellular processes and diseases, including cancer and associated drug resistance. Recent studies have highlighted the pivotal roles of circRNAs in mediating drug resistance mechanisms [18,29–31]. Huang et al. found that circSNX6 promotes sunitinib resistance through the circSNX6/miR-1184/GPCPD1 axis as a molecular “sponge” [29].

Additionally, CircPTK2 interacts with PABPC1 and enhances SETDB1 mRNA expression, thereby facilitating gemcitabine resistance in bladder cancer [32]. In hepatocellular carcinoma, circRNA-SORE overexpression is correlated with increased sorafenib resistance [33]. Furthermore, circUCP2 regulates the malignant behavior of non-small cell lung cancer through the miR-149/UCP2 pathway [34]. Mo et al. found that circMAN1A2 can sponge miR-940 and regulate ERBB2, which is associated with vasculogenic mimicry [35]. Emerging evidence has shown a relationship between circRNAs and sunitinib resistance in RCC [36–38]. In our previous studies, we found that circAGAP1 regulates ccRCC cell proliferation, migration, and invasion through other bypass or alternative pathways. It can act as a competing endogenous RNA (ceRNA) by interacting with miR-15a-5p, consequently blocking the inhibition of its target gene, E2F3. Furthermore, miR-15a-5p can regulate the malignant behavior of tumors by suppressing DLEU2 expression and modulating angiogenesis via VEGF-A inhibition. The function and mechanism of circAGAP1 in ccRCC needs to be further explored in both *in vitro* and *in vivo* studies.

In this study, we discovered that circAGAP1 expression was significantly lower in sunitinib-resistant cell lines than in sunitinib-sensitive cell lines and that it is functionally required for sunitinib-resistant phenotypes such as proliferation and migration. However, several questions remain unanswered. How does circAGAP1 regulate sunitinib sensitivity? Is circAGAP1 a molecular target for the regulation of sunitinib sensitivity? Based on the multi-target characteristics of sunitinib, combined with the report by Khella et al., miRNAs targeting VEGFR may be used as biomarkers to predict the remission rate of sunitinib [39]. Furthermore, our first study on the specific targeting and complementary binding of miR-15a-5p and circAGAP1 [27] showed that the target genes of miR-15a-5p include VEGFR2, the primary target of sunitinib action.

According to previous studies, ceRNAs have been linked to the development of drug resistance and tumor progression in a variety of tumor types. However, our study is one of the few to reveal that numerous circRNAs associated with sunitinib resistance are dysregulated in ccRCC tissues. Many studies have focused on a single miRNA; however, in this study, we found that circAGAP1 can act as a molecular sponge for multiple miRNAs, providing a more comprehensive explanation for the underlying mechanisms. We first identified miRNAs, including miR-149-5p, miR-455-5p, and miR-15-5p for which circAGAP1 acts as a sponge, and then predicted VEGFR and PDGFR to be potential target genes of miRNAs using bioinformatics analysis. Furthermore, this prediction was verified by dual-luciferase reporter and RNA Binding Protein Immunoprecipitation assays. Notably, PDGFR was the target gene of these miRNAs, whereas VEGFR was underexpressed in sunitinib-resistant RCC cells and was not considered for further research. Moreover, circAGAP1 may exert effects on ccRCC cells by mechanisms other than its ceRNA mechanism. These include modulation of parental gene expression, interaction with RNA-binding proteins, involvement in transcriptional regulation, and immune modulation within the tumor microenvironment. Each of these avenues offers a potential pathway through which circAGAP1 may effect ccRCC progression and patient outcomes. We acknowledge the need for further research to thoroughly explore these mechanisms.

Although our study provides an explanation for sunitinib resistance, some limitations still need to be noted. First, the results were obtained at the cellular level. However, owing to the complex regulatory mechanisms *in vivo*, our conclusions need to be validated *in vivo*. Second, due to the limited availability of clinical samples and time constraints, we were unable to expand our analysis within the desired timeframe. However, we are actively seeking collaborations to increase our sample size for future studies. Additionally, we intend to explore the clinical implications of our findings in subsequent studies to further validate and strengthen their clinical relevance. Emerging research indicates that circAGAP1 plays a significant role in renal clear-cell carcinoma progression. Targeting circAGAP1 offers a promising therapeutic avenue by potentially disrupting cancer-specific pathways [40]. Particularly, interventions that modulate circAGAP1 levels or interfere with its interaction with miRNAs may disrupt tumor-promoting pathways, leading to decreased tumor growth and metastasis. However, challenges include specific targeting and the minimization of off-target effects. Furthermore, a large number of samples, multiple centers, and independent external cohort validations are required for circAGAP1 to become a widely accepted biomarker.

Despite these challenges, the potential of circAGAP1 as a drug target for ccRCC remains promising. Advances in drug delivery systems, such as nanoparticle-based carriers (for example, Yuan et al. designed and synthesized a co-delivery nanosystem (Psc@DPP) that can adsorb platinum and si-circNUP5 with good capability to target medications and effectively treat diseases [41–43]), and the development of more specific and potent circAGAP1 modulators could overcome current limitations. Furthermore, elucidating the role of circAGAP1 in ccRCC progression and its interactions with other cancer-promoting mechanisms may reveal additional targets for combination therapies, thereby enhancing treatment efficacy and overcoming resistance.

## Conclusion

In summary, circAGAP1 regulates the expression of the sunitinib target PDGFR by acting as a miRNA sponge that simultaneously suppresses miR-149-5p, miR-455-5p, and miR-15a-5p. Overexpression of these three miRNAs reversed circAGAP1-mediated sunitinib sensitivity in ccRCC Identifying the multi-miRNA targets of circAGAP1 as a core hub molecule has become a breakthrough. Sunitinib therapy has not been fully explored in patients with RCC, largely because the absence of biomarkers obscures the identification of patients likely to respond to treatment. Our findings hold potential significance in predicting cancer prognosis and enhancing treatment strategies. Given their unique closed-loop structures, there is an urgent demand for reliable and consistent biomarkers that can effectively assess the clinical advantages of sunitinib treatment.

## Data Availability

All data are available from the corresponding author upon reasonable request.
